# Wnt signaling pathway in spinal cord injury: from mechanisms to potential applications

**DOI:** 10.3389/fnmol.2024.1427054

**Published:** 2024-07-24

**Authors:** Kai Li, Zanzhi Chen, Xuejing Chang, Ruiyang Xue, Huaibo Wang, Weitao Guo

**Affiliations:** Department of Spine Surgery, The Second Hospital Affiliated to Guangdong Medical University, Zhanjiang, China

**Keywords:** spinal cord injury, Wnt signaling pathway, clinical application, nerve regeneration, neuroinflammation

## Abstract

Spinal cord injury (SCI) denotes damage to both the structure and function of the spinal cord, primarily manifesting as sensory and motor deficits caused by disruptions in neural transmission pathways, potentially culminating in irreversible paralysis. Its pathophysiological processes are complex, with numerous molecules and signaling pathways intricately involved. Notably, the pronounced upregulation of the Wnt signaling pathway post-SCI holds promise for neural regeneration and repair. Activation of the Wnt pathway plays a crucial role in neuronal differentiation, axonal regeneration, local neuroinflammatory responses, and cell apoptosis, highlighting its potential as a therapeutic target for treating SCI. However, excessive activation of the Wnt pathway can also lead to negative effects, highlighting the need for further investigation into its applicability and significance in SCI. This paper provides an overview of the latest research advancements in the Wnt signaling pathway in SCI, summarizing the recent progress in treatment strategies associated with the Wnt pathway and analyzing their advantages and disadvantages. Additionally, we offer insights into the clinical application of the Wnt signaling pathway in SCI, along with prospective avenues for future research direction.

## 1 Introduction

Spinal Cord Injury (SCI) pertains to the impairment of spinal cord structure and function due to external trauma or internal pathological factors. As a component of the central nervous system nestled within the spinal canal, the spinal cord facilitates the transmission of sensory and motor signals between the brain and various bodily regions. SCI can lead to partial or complete loss of sensory and motor functions in specific body regions, potentially accompanied by dysfunction of the autonomic nervous system (Tarvonen-Schröder et al., 2018). This not only inflicts severe physical and psychological damage upon the affected individuals but also imposes a substantial economic burden on society (GBD Spinal Cord Injuries Collaborators, 2023). Currently, the global annual incidence of SCI is estimated to be between 40 to 80 cases per million population, with statistical data indicating that the majority of SCI cases occur in young adults, constituting approximately 80% of those below the age of 40. Gender-specific incidence rates vary across regions, yet the overall male incidence rate of SCI remains higher. The male-to-female ratio of SCI incidence ranges from 1.6:1 to 8:1 (Ding et al., 2022). Annually, approximately 250,000 to 500,000 individuals worldwide suffer from SCI, with up to 90% of cases resulting from traumatic causes, such as traffic accidents, falls, or acts of violence (Thietje et al., 2011; GBD Spinal Cord Injuries Collaborators, 2023).

**Figure 1:**
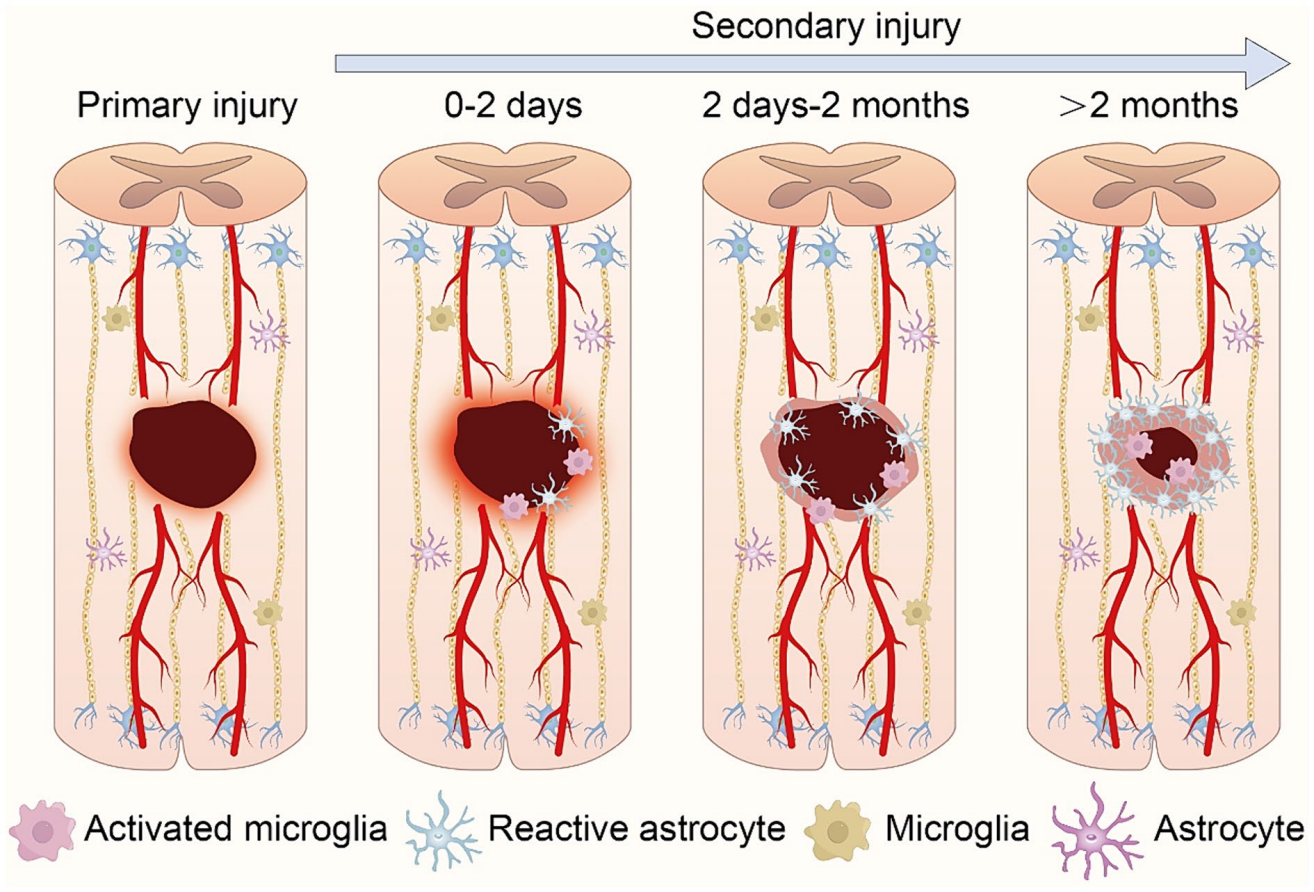
Schematic representation of the pathophysiological processes following spinal cord injury. Following primary injury, local bleeding and necrosis occur at the wound site. In secondary injury, central immune cells aggregate and activate, inducing further expansion of the injury zone. As the pathology advances, primary glial scars form, isolating inflammation. Ultimately, glial scars mature, accompanied by central infiltrative inflammation.

SCI are categorized into primary and secondary types, each characterized by distinct features and developmental trajectories. Primary SCI refers to the immediate damage to the spinal cord incurred during traumatic events such as accidents, falls, or sports injuries. This type of injury occurs instantaneously at the moment of trauma and is typically caused by mechanical forces such as compression, stretching, or torsion exerted on the spinal cord, leading to nerve fiber rupture, spinal cord cell damage, and vascular rupture. Secondary SCI, encompasses the subsequent injury progression and functional impairments following the primary injury. Secondary injury often arises from inflammation, cellular swelling, and lipid oxidation triggered by the primary injury. These inflammatory and cellular damages may exacerbate the initial SCI, resulting in further neuronal death and functional loss (Alizadeh et al., 2018; Sterner and Sterner, 2023; Figure 1).

The pathological mechanisms underlying SCI are remarkably intricate, involving numerous signaling pathways such as the Wnt signaling pathway, NF-κB signaling pathway, Hedgehog signaling pathway, PI3K/Akt signaling pathway, and Janus kinase/signal transducer and activator of transcription (JAK/STAT) pathway (Ge et al., 2021; Hamilton et al., 2021; Wang et al., 2023; Gao et al., 2024; Zhao et al., 2024). Among these, the Wnt signaling pathway contributes to processes including neurodevelopment, cell proliferation, and fate determination of neural stem cells, playing a crucial role in the pathological changes of central nervous system diseases (Xu et al., 2022). Extensive research reports underscore the pivotal role of the Wnt signaling pathway in SCI. In the early stages of SCI, ischemia and necrosis are the primary events, where the Wnt signaling pathway promotes neuronal regeneration under hypoxic conditions (Lu P. et al., 2019; Chen M. et al., 2023). During the oxidative stress and neuronal apoptosis phases, the Wnt signaling pathway enhances neuronal survival (Yang W. et al., 2022). In the chronic phase of SCI, the Wnt signaling pathway participates in scar formation, axonal regeneration, and neural network reconstruction (Chen T. et al., 2024). Additionally, the Wnt signaling pathway gaining prominence for its involvement in regulating inflammation, cellular apoptosis and survival, as well as neural regeneration and repair (Zhang M. et al., 2020; Gao et al., 2024). For example, CHIR99021 is a drug that selectively inhibits GSK3β to stabilize β-catenin and promote Wnt signaling transmission (Chambers et al., 2009). Studies have found that CHIR99021 promotes the reprogramming of neural glial cells and stem cells into neurons, thereby revealing the direct impact of Wnt signal modulators on neuronal repair without introducing exogenous genetic factors. Subsequent *in vivo* experiments further validate the efficacy of these results (Li et al., 2015; Tan et al., 2024). Overall, the Wnt signaling pathway demonstrates substantial potential in SCI treatment.

However, the Wnt signaling pathway may also lead to adverse effects. For example, excessive activation of the Wnt pathway can cause an overreaction of astrocytes, leading to the formation of extensive glial scars that impede axonal regeneration and functional recovery (Sareddy et al., 2009). In some cases, Wnt pathway activation may promote the release of inflammatory factors, exacerbating inflammation and neuronal damage (An et al., 2024). Overactivation of the Wnt signaling pathway might also result in abnormal proliferation and differentiation of neural stem cells, leading to tumor formation or other pathological tissues (Yang Y. et al., 2022). Therefore, it is imperative to continue researching its mechanisms and develop safe and effective regulatory methods to fully harness its therapeutic potential.

In conclusion, SCI remains a major threat to human health, characterized by high incidence, severe clinical manifestations, and poor prognosis, making it a significant challenge in the medical field. With ongoing research, scientists have recognized the crucial role of the Wnt signaling pathway in neural regeneration and repair following SCI. This review highlights the latest advancements and potential therapeutic strategies involving both canonical and non-canonical Wnt signaling pathways in SCI. Additionally, it discusses the clinical applications of the Wnt signaling pathway in SCI and explored future research directions. The goal of this review is to offer novel and valuable approaches for the restoration of neurological function following SCI.

## 2 Wnt singling pathway

The Wnt signaling pathway maintains core functionality and mechanism conservation across various species. This conservation ensures the pathway’s critical role in regulating fundamental biological processes such as cell proliferation, differentiation, migration, and survival, while allowing different species to adaptively regulate the pathway according to their specific biological requirements (Xu et al., 2022). Based on whether it relies on β-catenin for signal transduction to regulate downstream gene expression, it is classified into canonical and non-canonical pathways (Albrecht et al., 2021; Cooney et al., 2023). The canonical Wnt signaling pathway relies on Wnts to initiate the degradation of the β-catenin complex, facilitating cascades of signal transduction and subsequently regulating cellular physiological activities. In contrast, the non-canonical Wnt signaling pathway does not involve β-catenin in signal transduction.Their primary signaling routes include the Wnt/planar cell polarity (PCP) pathway and the Wnt/Ca^2+^ pathway, which play crucial roles in regulating cytoskeletal organization, cell movement, cell polarity, and calcium ion signaling (Bell et al., 2022; Rim et al., 2022).

Currently, 19 different Wnt proteins, 10 Frizzled receptors (FZDs), 5 secreted Frizzled-related proteins (Sfrps), and 4 Dickkopfs (DKKs) have been identified (Ring et al., 2011). Generally, Wnt ligands (Wnt1, Wnt2, Wnt2b, Wnt3, Wnt3a, Wnt7a, Wnt7b, Wnt8a, Wnt8b, Wnt9a, Wnt9b, Wnt10a, Wnt10b) and Wnt receptors (FZD1, FZD4, FZD7, FZD8) mediate the canonical Wnt signaling pathway. Wnt4, Wnt5a, Wnt5b, Wnt6, Wnt11, Wnt16, FZD2, FZD3, FZD6, FZD10, ROR1/2, and RYK participate in the non-canonical Wnt signaling pathway transmission (Sonavane and Willert, 2021; De Almeida et al., 2024). Secreted Frizzled-related proteins (sFrps) (sFrp1-5) and Dickkopfs (DKK1-4) are common inhibitors of the Wnt signaling pathway. Notably, sFrps and FZDs compete to bind Wnt ligands to inhibit Wnt signal transduction, while DKKs interact with the auxiliary receptor low-density lipoprotein receptor-related protein 5/6 (LRP5/6) to inhibit Wnt/β-catenin signal transduction (Kawano and Kypta, 2003).

**Figure 2:**
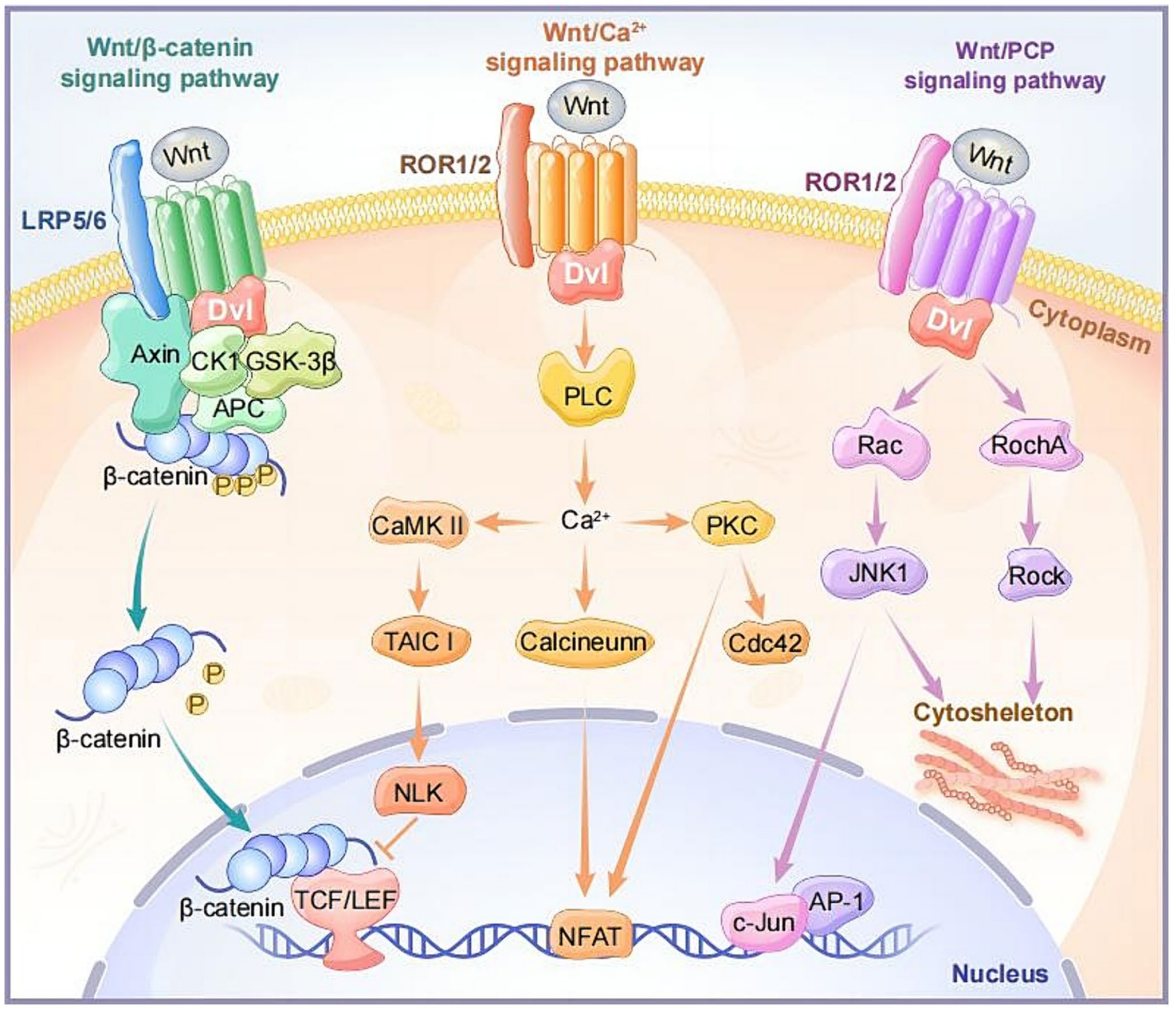
The intracellular components and signaling transduction of canonical and non-canonical Wnt signaling pathways.

In the canonical Wnt signaling pathway, when Wnt ligands do not bind to receptors, intracellular GSK3β and ck1α induce phosphorylation of β-catenin, making phosphorylated β-catenin more prone to ubiquitination and degradation in the cytoplasm (Shah and Kazi, 2022). When Wnt ligands bind to FZD receptors, along with the co-receptor LRP5/6, they recruit cytoplasmic Disheveled (DVL) around FZD. Subsequently, the conformation of DVL changes, triggering the phosphorylation of LRP5/6 (Qi et al., 2017). AXIN is recruited to phosphorylated LRP5/6, collectively inhibiting GSK3β phosphorylation activation. This facilitates the disintegration of the AXIN-GSK3β-APC-β-Catenin complex, leading to an increase in cytoplasmic β-Catenin concentration, subsequently entering the cell nucleus. There, it binds to T-cell factor/lymphoid enhancer factor (TCF/LEF) and recruits transcriptional co-activators such as cAMP response element-binding (CREB) and CREB-binding protein (CBP), forming an active transcription complex that activates downstream target gene expression including *Axin2, c-Myc, Cyclin-D1 (Ccdn1), Cd44, Mmp2/9, and vegf* (Zeng et al., 2018; Hayat et al., 2022; Figure 2).

The Wnt/Ca^2+^ signaling pathway is a non-classical Wnt signaling pathway that leads to an increase in intracellular calcium ion (Ca^2+^) concentration through specific signaling molecules and steps. Unlike the classical Wnt/β-catenin pathway, this pathway does not depend on the stability of β-catenin and its transcriptional activity (Wang et al., 2024). Upon binding of Wnt ligands (Wnt4, Wnt5a, Wnt5b, Wnt6, Wnt7a, Wnt11, etc.) and receptors FZDs, the activation of the DVL leads to the activation of heterotrimeric G proteins, further activating phospholipase C (PLC). This induces a transient increase in intracellular calcium ion concentration, and Ca^2+^ induces the activation of calcium-calmodulin-dependent kinase (CaMKII) and protein kinase C (PKC). Notably, the activation of CaMKII stimulates the TAK1-NLK pathway, inhibiting gene expression induced by wnt/β-catenin in the cell nucleus (Abdolmaleki et al., 2020; Yu et al., 2023; Figure 2).

The non-canonical planar cell polarity pathway, Wnt/PCP, is involved in regulating cell polarity and directional growth. Wnt ligands (Wnt5a, Wnt5b, Wnt11, etc.) can activate DVL when binding to receptors FZD, ROR, or Ryk. Activated DVL participates in the activation of small GTPase proteins RhoA and Rac, subsequently activating stress kinases JNK and ROCK. This results in the formation of the cell skeleton and cell adhesion and movement, regulating the asymmetric distribution and polarization of cells (Vivancos et al., 2009; Nagaoka et al., 2023; Figure 2).

## 3 Role of the Wnt signaling pathway in SCI

### 3.1 Variations in Wnt pathway expression among species in SCI

SCI represents a complex molecular-mediated pathological process, exhibiting distinct pathophysiological responses across different species and genders (Stewart et al., 2021; Zrzavy et al., 2021). For instance, rats and mice demonstrate varying degrees of occurrence and magnitude of lymphocyte and dendritic cell infiltration following SCI (Sroga et al., 2003). In contrast to rodents, the degree of lymphocyte infiltration in the injured spinal cord of humans is notably less during both the acute and chronic phases of SCI (Zrzavy et al., 2021). Inflammatory responses in rodents tend to be more intense, characterized by significant infiltration of inflammatory cells and release of inflammatory factors. Conversely, human inflammatory responses may be relatively subdued but prolonged. Additionally, rodents may demonstrate more pronounced nerve regeneration and tissue reconstruction following SCI, whereas humans often exhibit limited nerve regeneration capacity, leading to the formation of scar tissue and hindering effective tissue regeneration. These differences can be attributed to various factors, including physiological structures, immune system responses, gene expression, and environmental influences (Dietz and Schwab, 2017; Han et al., 2019). Given its role as a key molecular pathway regulating neuroinflammation and neuroregeneration following SCI, it is not surprising to observe differential expression of the Wnt signaling pathway across different species.

With the advancement of research, scientists have found that in rats, zebrafish, and salamanders, the Wnt signaling pathway is widely activated in the tissues post-SCI (Gonzalez et al., 2012; Ponomareva et al., 2015; Fu et al., 2019). In these model organisms, the activation of the Wnt signaling pathway may be involved in processes such as proliferation and differentiation of neural progenitor cells, neuronal anti-apoptosis, activation of oligodendrocytes, and axonal regeneration following SCI. In contrast, mice exhibit an opposing expression pattern of the Wnt signaling pathway. Following SCI in mice, the sustained upregulation of Wnt inhibitory factor 1 (Wif1) expression and the continuous downregulation of Wnt3a expression imply a progressive increase in neuronal death. Specifically, the prominent peak of Wif1 upregulation in mice after SCI coincides with the peak of neuronal death, precisely illustrating the crucial role of the Wnt signaling pathway in neuronal survival following SCI (González-Fernández et al., 2014). These data highlight the crucial role of the Wnt protein family in both healthy and damaged spinal cords, and the genes with differentially expressed patterns could potentially serve as therapeutic targets post-SCI. However, further research is needed to elucidate the expression patterns and molecular mechanisms of all Wnts and their mediated pathways in various cell types in SCI.

**Figure 3:**
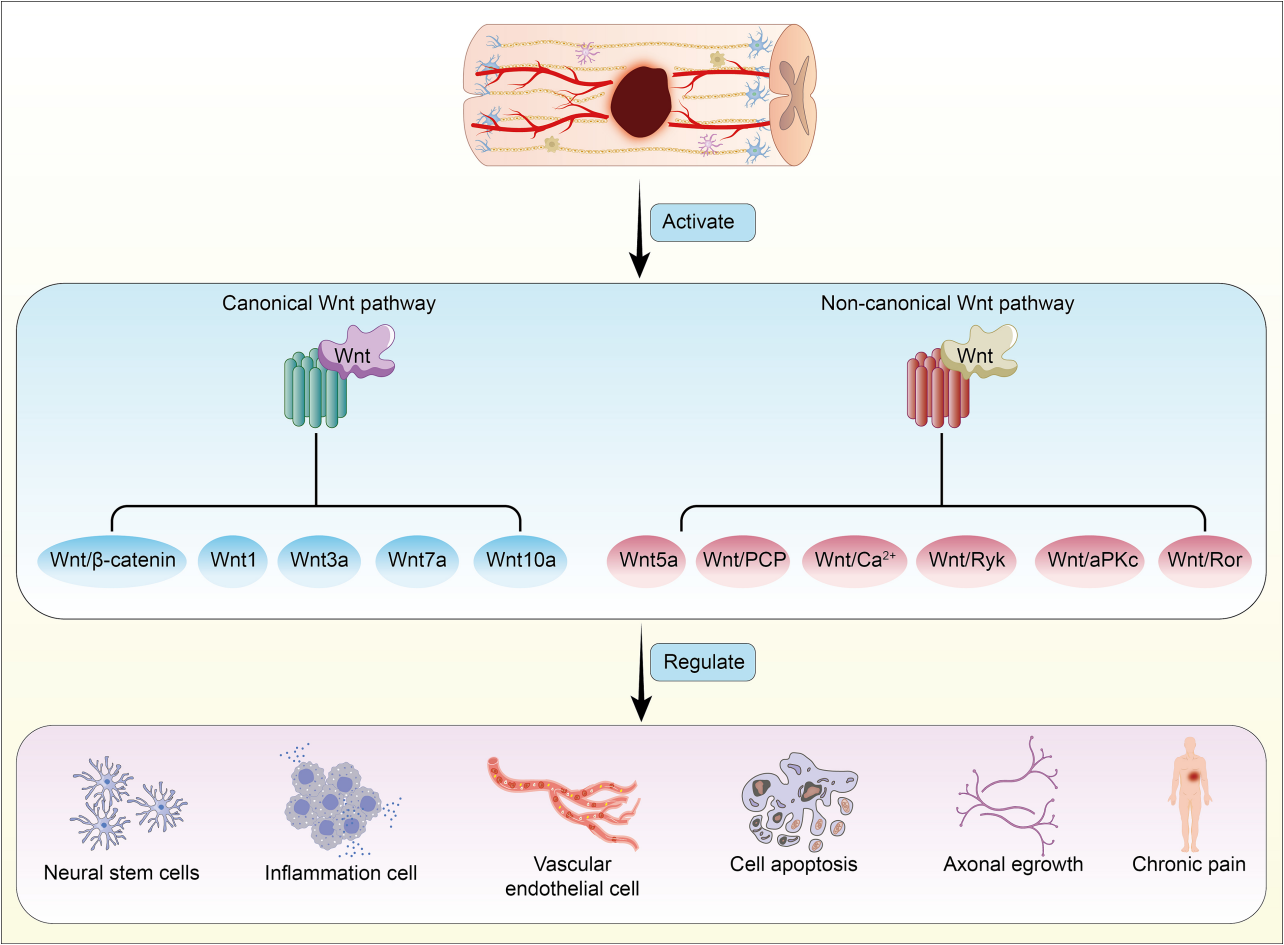
Activation of canonical and non-canonical Wnt signaling pathways following spinal cord injury, mediated by different Wnt proteins, leads to reciprocal regulation and induces pathophysiological changes in the body.

Here, we have summarized six research directions with potential application value concerning the Wnt signaling pathway in SCI (Figure 3), and we provide an overview of the mechanisms and functions of different Wnt proteins on target organs (Table 1).

**Table 1:** Mechanisms and effects of different Wnt proteins on various target organ.

Pathway types	Wnt molecular	target organs	Mechanisms and functions
Canonical Wnt signaling pathways	Wnt1	Inflamation cell	Inducing microglial polarization to the M2 phenotype inhibits the inflammatory response.
	Wnt/β-catenin	Neural stem cells	Promote the proliferation and differentiation of other stem cell types into neuron-like cells
		Inflamation cell	Inhibit microglial activation to protect spinal cord neurons
		Vascular endothelial cell	Promote the generation and maturation of endothelial cells
		Cell apoptosis	Inhibit apoptosis to protect spinal cord neurons
		Axonal egrowth	Induce morphological changes in astrocytes to activate axonal regeneration
	Wnt3a	Neural stem cells	Promote proliferation and differentiation of neural stem cells while inhibiting apoptosis
		Inflamation cell	Inducing microglial polarization to the M2 phenotype inhibits the inflammatory response
		Cell apoptosis	Inhibit autophagy in motor neurons to reduce cell apoptosis
		Axonal egrowth	Induce differentiation of oligodendrocyte precursor cells to promote remyelination.
		Chronic Pain	Induce activation of astrocytes to promote release of inflammatory cytokines, leading to pain
	Wnt7a	Vascular endothelial cell	Promote blood-spinal cord barrier repair
		Chronic Pain	Alleviate hyperalgesia and neuropathic pain following spinal cord injury
	Wnt10a	Chronic Pain	Induce astrocyte activation leading to pain hypersensitivity
Non-canonical Wnt signaling pathways	Wnt5a	Neural stem cells	Promote synaptic growth following spinal cord injury
		Inflamation cell	Induce inflammation activation of microglial cells
		Vascular endothelial cell	Promote blood-spinal cord barrier repair.
		Chronic Pain	Elevating chronic inflammation levels and leading to hyperalgesia
	Wnt/PCP	Cell apoptosis	Trigger cell cytoskeleton reorganization and stress response, inducing apoptosis
	Wnt/Ca2+	Cell apoptosis	Activating calcium-dependent effector molecules leads to cell apoptosis
	Wnt/Ryk	Vascular endothelial cell	Causing changes in vascular endothelial cell morphology and increased permeability
		Chronic Pain	Elevating chronic inflammation levels and leading to hyperalgesia
		Axonal egrowth	Inhibiting axonal growth following spinal cord injury
	Wnt/PKc	Axonal egrowth	Causing retraction of corticospinal tract axons from the lesion site and inhibiting growth of proximal axon segments.
	Wnt/Ror	Axonal egrowth	Involved in neuronal migration, extension, and axon pruning.
		Chronic Pain	Elevating chronic inflammation levels and leading to hyperalgesia

### 3.2 Induction of proliferation and differentiation of neural stem cells

By inducing the proliferation and differentiation of stem cells to replace damaged cells, tissue repair is the most effective mode of the body’s response to injury. The Wnt signaling pathway is involved in the proliferation and differentiation of neural stem cells in the brain and spinal cord (Willert et al., 2003). Canonical and non-canonical Wnt signaling pathways are involved in the proliferation and differentiation of spinal cord neural stem cells, playing distinct roles in SCI (Chenn and Walsh, 2002; Yun et al., 2007).

In adult spinal cord tissue, Wnts expression are scarcely expressed. However, during SCI, Wnt1 plays a pivotal role in orchestrating the activation of ependymal cells from their quiescent state. This activation drives ependymal cell proliferation, which in turn contributes to SCI repair, ensuring the stability of the spinal cord environment and the structural integrity of the injured site (Shinozuka et al., 2019). The canonical Wnt signaling pathway mediated by Wnt3a also plays a crucial role in inducing proliferation and differentiation of neural stem cells. Activation by Wnt3a enhances the proliferative capacity of neural stem cells, typically by increasing the number of cells in the S phase of the cell cycle, thereby expanding the neural stem cell population (Wang et al., 2023). Wnt3a promotes differentiation of neural stem cells toward a neuronal lineage, potentially through modulation of specific gene expression patterns such as neurogenic markers (e.g., NeuroD1,β-tubulin; Somredngan et al., 2023). Furthermore, following SCI, Wnt3a can significantly inhibit neuronal apoptosis and inflammatory responses, reduce the loss of motor neurons in the anterior horn of the spinal cord, and promote the repair of damaged tissue. This creates a favorable microenvironment for the restoration of motor neuron function, ultimately facilitating the recovery of motor function (Li W. et al., 2023; Gao et al., 2024). Wnt/β-catenin signaling transduction significantly enhances the proliferative capacity of sensory interneurons, preserving their ability to differentiate into specific sensory neurons as needed (Gupta et al., 2022). It has also been reported that activating Wnt/β-catenin can promote the proliferation and differentiation of other types of stem cells into neuron-like cells. When the Wnt pathway is inhibited, the proliferation and differentiation of neuron-like cells are eliminated (Hu et al., 2019).

Both the canonical and non-canonical Wnt signaling pathways play crucial roles in neurogenesis and neuronal maturation. Research indicates that GDE2, a canonical Wnt signaling transducer, regulates a neuronal pathway that signals to oligodendrocytes, promoting their maturation (Choi et al., 2020). In another study of the non-canonical Wnt pathway, BML-281 was found to induce the differentiation of SH-SY5Y cells into mature neurons by activating this pathway. BML-281 is suggested as a novel drug target that regulates the non-canonical Wnt signaling pathway to reduce neuronal cell death and promote differentiation into neurons (Choi et al., 2023). The non-canonical signaling pathway mediated by Wnt5a demonstrates a promoting role in neurite outgrowth in spinal cord-derived neural stem/progenitor cells (NSPCs). Additionally, it may facilitate functional recovery and repair of the nervous system by potentially inhibiting the accumulation of astrocytes (Zhao B. et al., 2021).

In summary, the canonical Wnt signaling pathway primarily promotes functional recovery and injury repair of neural stem cells through regulation of cell proliferation, differentiation, and self-renewal abilities. In contrast, the non-canonical Wnt signaling pathway may be more involved in processes such as cell polarity regulation and cell migration, which are equally crucial for neural stem cell differentiation and neuroregeneration. Therefore, in the treatment of SCI, a comprehensive consideration and modulation of the activities of these two signaling pathways may represent an effective therapeutic strategy. This approach aims to enhance the proliferation and differentiation of neural stem cells, thereby promoting repair and functional recovery of the nervous system.

### 3.3 Activated microglia regulate the inflammatory response

After SCI, the inflammatory response occurs within minutes and can persist for several days, months, or even years (Wichmann et al., 2022). Microglials are a type of specialized mononuclear phagocyte originating from the embryonic yolk sac, maturing within the central nervous system (Ginhoux and Prinz, 2015). The activation of microglia is a crucial hallmark of the inflammatory response in SCI. As intrinsic immune cells in spinal cord tissue, microglia can differentiate into various subtypes and play essential roles in the inflammatory response at different stages of SCI (Milich et al., 2012). Research indicates the involvement of the Wnt signaling pathway in the activation of microglia in SCI (Van Steenwinckel et al., 2019). In SCI, different subtypes of microglials undergo dynamic changes. The M1 subtype can release interleukin-6 (IL-6), interleukin-1β (IL-1β), tumor necrosis factor alpha (TNF-α), thereby promoting inflammation. Conversely, the M2 subtype plays a crucial role in immune regulation and neural regeneration (Xu et al., 2018). Studies have found significant differences in the expression of Wnts in different subtypes of microglia and macrophages, indicating an association between Wnts and the activation status of various microglial subtypes (González et al., 2022). M1-polarized microglials release Wnt5a, promoting inflammation. Conversely, M2-polarized microglials secrete Wnt7a, wherein Wnt7a/β-catenin plays a crucial role in the differentiation of microglials progenitor cells and neural repair (Mecha et al., 2020). The non-canonical Wnt signaling pathway mediated by Wnt5a can induce inflammatory activation of microglials, thereby increasing the infiltration area of microglials (Jiangxue et al., 2023). Wnt1 and Wnt3a induce the transformation of microglials into an M2 phenotype, suppressing inflammatory responses, and promoting neural repair (Matias et al., 2019; Gao et al., 2022). Generally, the activation of the non-canonical Wnt signaling pathway promotes microglial activation and induces an inflammatory response, while the activation of the canonical Wnt signaling pathway inhibits microglial proliferation and alleviates inflammation.

In the early stages of SCI, the upregulation of inflammation is beneficial for clearing damaged cells and stimulating neuronal regeneration, providing neuroprotection. Research has shown that SIRT1 may have a neuroprotective effect by suppressing microglial activation via downregulation of the Wnt/β-catenin signal following SCI (Lu T. et al., 2019). Microglia can also secrete Wnt5a to stimulate neuronal growth and synapse structure maturation (Yeh et al., 2023). In the subacute phase of SCI, M2 macrophages release Wnt3a, inducing the migration and activation of astrocytes, participating in the formation of neuroglial scars, effectively limiting the spread of inflammation and preventing the enlargement of the inflammatory area into the surrounding tissue (Sonn et al., 2020). Therefore, the relationship between the Wnt signaling pathway and microglial inflammation is complex, and its specific role may change with time, injury type, and environmental factors. Furthermore, studies have found that depletion of microglia following SCI disrupts the formation of glial scars, enhances immune cell infiltration, reduces neuronal survival, and impairs neural function recovery (Fu et al., 2020). Considering the impact of Wnt signaling on microglia, depleting Wnt signaling pathway members in microglia may potentially lead to uncontrolled inflammation, reduced neuroprotective effects, diminished repair capabilities, and increased cell death, thereby exacerbating the pathological state after spinal cord injury and affecting the recovery process. However, the specific mechanisms underlying these outcomes are currently unclear, which could serve as a valuable direction for future exploration.

### 3.4 Induction of vascular neogenesis and stability

The actue SCI results in local vascular damage and disruption of the blood-spinal cord barrier (BSCB), leading to increased vascular permeability. These changes not only induce spinal tissue ischemia and hypoxia but also exacerbate inflammatory responses, triggering cellular apoptosis, among other consequences (Ge et al., 2021). Thus, maintaining vascular integrity is crucial for sustaining the balance of the blood-spinal fluid microenvironment.

Under both normal and pathological conditions, canonical Wnt and non-canonical Wnt signals play pivotal roles in angiogenesis (Lin et al., 2024). During development, Wnt5a/PCP participates in the polarization of endothelial cells, promoting the formation of tight junctions (Langford et al., 2020). The high expression of β-catenin in endothelial cells transforms them into a high-permeability state, contributing to the barrier-type status (Wang et al., 2018; Wang Y. et al., 2019). Therefore, the Wnt signaling pathway plays a crucial role in the formation and maturation of the BSCB. In central nervous system injuries, endothelial cell Wnt/β-catenin signal transduction stimulates angiogenesis and maturation (Chavali et al., 2020). In models of neural injury, the inactivation of the Wnt/β-catenin signaling pathway may be a cause of early blood vessel barrier disruption (Moreau et al., 2017). Additionally, Wnt7a and Wnt5a have been shown to participate in the repair of BSCB damage in amyotrophic lateral sclerosis (Ouali Alami et al., 2020). However, whether the Wnt signaling pathway is involved in BSCB repair after SCI remains to be investigated.

The generation of new blood vessels can provide substantial nutritional support for the homeostasis of neuronal networks post-SCI, and regenerated blood vessels can serve as a scaffold for axonal growth (Rauch et al., 2009). Simultaneously, appropriately increasing spinal cord vascular density can promote functional recovery after SCI (Widenfalk et al., 2003). Exogenous induction of Wnt/β-catenin reactivation can stimulate endothelial cells to secrete vascular growth factors such as VEGF, IL-8, Cyclin D1, and DLL4, effectively regulating vascular regeneration and neural functional recovery after SCI (Luo et al., 2021). Concerning the non-canonical Wnt signaling pathway, SCI induces the activation of macrophages and the secretion of Wnt5a (González et al., 2021). However, the activation of endothelial cell Wnt5a/Ryk induces changes in cell morphology and increased permeability (Skaria et al., 2017). Meanwhile, macrophages express the effective anti-angiogenic molecule VEGF-R1 through an internal non-canonical Wnt-dependent pathway, thereby limiting abnormal or excessive angiogenesis (Stefater et al., 2013). Thus, the non-canonical Wnt signaling pathway holds exceptional significance in tissue vascular neogenesis and vascular structural remodeling. Inducing the activation of both canonical Wnt and non-canonical Wnt signaling pathways after SCI has positive implications for maintaining BSCB stability and promoting vascular neogenesis post-SCI.

### 3.5 Inhibition of neuronal apoptosis

Cell apoptosis induced by SCI occurs within hours to weeks and is a significant factor contributing to the expansion of damage and hindering neural recovery (Kuzhandaivel et al., 2011). SCI-induced cell apoptosis can be categorized into endogenous apoptosis and exogenous apoptosis. The primary mechanical injury to cells at the site of SCI activates their endogenous apoptotic pathways when these cells sustain sufficient damage. In contrast, surrounding neural cells undergo exogenous cell apoptosis due to local ischemia and oxidative stress reactions induced by injury (He et al., 2023b).

Research has revealed that the Wnt signaling pathway regulates neuronal apoptosis through various mechanisms, including the Wnt/β-catenin signaling pathway and Wnt activation of the BMP or NF-κB signaling pathways (Pinto et al., 2013; Tiong et al., 2019; Ozalp et al., 2021). In both *in vivo* and *in vitro* models of traumatic SCI, activation of the Wnt/β-catenin signaling pathway suppresses the expression of apoptosis proteins Bax, caspase-9, and caspase-3 (He et al., 2023a). The circRNA/miRNA/mRNA network plays a role in the physiological and pathological processes of SCI (Wang et al., 2021; Li Y. et al., 2022). MiR-137 is a crucial molecule in delaying neuronal apoptosis. It reduces neuronal apoptosis not only through the Src/MAPK and JAK/STAT1 axes but also by downregulating apoptosis levels through the KDM4A/SFRP4/Wnt3a/β-catenin axis (Tian et al., 2020; Zhang T. et al., 2020; Li Y. et al., 2023). Circ-Ctnnb1 activates the miR-205-5p/Ctnnb1/Wnt2a/β-catenin signaling pathway, suppressing neuronal apoptosis (Qi et al., 2022). MiR-381 induces the activation of the BRD4/Wnt5a axis in dorsal root ganglion cells, inhibiting apoptosis and rescuing injured neurons (Jia et al., 2021).

The non-canonical Wnt signaling pathway also plays a crucial role in promoting apoptosis. Researchers have found that the knockdown of CaMKII, a downstream target of the non-canonical Wnt pathway, can inhibit apoptosis (Zhang et al., 2015). Activation of the Wnt/PCP signaling pathway can lead to cytoskeletal reorganization and stress responses, potentially inducing apoptosis by affecting cell survival signals (Hu et al., 2020; Qin et al., 2024). Furthermore, after SCI, intracellular calcium levels may increase, and through the Wnt/Ca^2+^ signaling pathway, various calcium-dependent effector molecules are activated.These molecules can regulate apoptosis-related gene expression, thereby inducing cell apoptosis (Liu J. et al., 2022; Wang et al., 2024). It is noteworthy that apoptosis in SCI is closely related to autophagy, which can exert neuroprotective effects by inhibiting cell apoptosis in acute SCI rats. Wnt3a in the SCI region inhibits mTOR-mediated autophagy in motor neurons to reduce apoptosis, preserving surviving neurons (Gao et al., 2020). Therefore, the regulation of cell apoptosis by the Wnt signaling pathway is a crucial mechanism in adapting to the microenvironment of SCI and maintaining cellular homeostasis.

### 3.6 Promotion of axonal growth

Following SCI, local axons and synapses of neurons are damaged. Over time, the injured neural axons undergo complete degeneration in the distal segments, while the proximal parts retract over a relatively short distance. This phenomenon hinders the regeneration of neuronal axons and the reconstruction of functional connections. Wnt was initially discovered as a axon guidance molecule, directing neural axons to grow along the anterior–posterior axis of vertebrates (Hua et al., 2014; Wang Y. et al., 2022). The Wnt signaling pathway is involved in the formation and function of synaptic structures. In adulthood, as the nervous system matures and stabilizes, the expression level of the Wnt signaling pathway in axons significantly decreases. However, in the early stages of SCI, neurons and glial cells surrounding the injury site release cytokines, including Wnt proteins. These Wnt proteins can activate the Wnt signaling pathway on receptor cells to promote axonal regeneration and synaptic reconstruction. Upregulation of the Wnt signaling pathway can enhance axonal growth and regeneration by regulating the expression of axon guidance molecules (McLeod et al., 2018). However, the impact of Wnt ligands on axonal function is complex, as their concentration-dependent effects, when binding to different receptors, can lead to opposing activities.

In SCI, canonical Wnt signaling transduction can induce axonal regeneration and neurite outgrowth. Wnt3a in the SCI zone can induce the differentiation of oligodendrocyte precursor cells, promoting myelin sheath repair in axonal injuries (Yin et al., 2008). Simultaneously, the Wnt/β-catenin pathway in fibroblast-like cells in the injury zone is reactivated, inducing changes in the morphology of astrocytes and activating axonal regeneration through the secretion of regenerative collagen XII (Wehner et al., 2017). However, the inhibition of the Wnt/β-catenin signaling pathway severely diminishes axonal regeneration in the damaged spinal cord and the formation of glial bridges, impeding functional improvement in motor skills (Strand et al., 2016).

Conversely, activation of the Wnt/Ryk signaling pathway is detrimental to axonal regeneration. Local activation of the Wnt/Ryk signaling transduction system in SCI causes the retraction of corticospinal tract axons from the lesion and inhibits the growth of proximal axon segments (Liu et al., 2008). Massive activation of the non-canonical pathways Wnt/Ryk and Wnt/aPKC in motor neurons induces the loss of axonal growth function (Tury et al., 2014). Despite the non-canonical Wnt signaling pathway being unfavorable for axonal regeneration after SCI, it plays a crucial role in neural circuit reconstruction post-SCI (Simonetti and Kuner, 2020). For example, the wnt5a/Ryk pathway is deemed essential for the proper formation of neuromuscular junctions (Liebl et al., 2008). The Wnt/Ror pathway is expressed in neural circuits and is involved in processes such as neuronal migration and extension, as well as axonal pruning (Kennerdell et al., 2009; Peysson et al., 2024). The balance between the canonical and non-canonical Wnt signaling pathways is pivotal for axonal repair after SCI.

### 3.7 Induction of chronic pain

Chronic neuropathic pain following SCI has been a complex condition, characterized not only by multiple potential pathophysiological mechanisms but also linked to sociopsychological factors. Studies have revealed an upregulation of both canonical and non-canonical Wnt signaling pathways in SCI-induced neuropathic pain and the dorsal horn of the spinal cord (Shi et al., 2012; Yuan et al., 2012; Wang et al., 2021). The implicated mechanisms involve the upregulation of the Wnt signaling pathway, leading to increased inflammation levels in neurons and inducing the reshaping and amplification of synaptic connections, thereby magnifying pain signaling (Ru et al., 2019; Lu et al., 2023). Research has identified the activation of non-canonical signaling pathways in the spinal dorsal horn, such as Wnt5a/Ryk/Ror2, Wnt5a/CaMKII/NFAT, and Wnt5a/ROR2/MMP2, which elevate chronic inflammation levels and contribute to hyperalgesia (Simonetti et al., 2020; Liu X. et al., 2022; Lu et al., 2023). Components of the canonical signaling pathway, including Wnt3a, Wnt10a, and β-catenin, exhibit increased expression levels associated with chronic neuropathic pain (Kim et al., 2021). Wnt3a has been found to stimulate the activation of spinal astrocytes in a neuropathic pain model, leading to the release of pro-inflammatory cytokines TNF-α and IL-18. Simultaneously, Wnt10a/β-catenin is involved in kindlin-1 mediated astrocyte activation during the later stages of SCI. Knockdown of Wnt10a can reduce hyperalgesia and allodynia following SCI (Kim et al., 2021; Zhao C. G. et al., 2021). However, the expression patterns of canonical and non-canonical Wnt pathways in the pathological process of chronic pain differ, likely involving distinct biological functions (Shi et al., 2012). Further exploration of the relationship between different Wnt signaling pathways and the induction of chronic pain post-SCI will aid in the development of therapeutic targets for chronic pain.

## 4 Treatment

Within the context of SCI, the Wnt signaling pathway plays a crucial role in inducing the proliferation and differentiation of neural stem cells, regulating levels of inflammation and apoptosis, promoting vascular neogenesis and repair, as well as axonal regeneration and repair. The evident clinical potential of the Wnt signaling pathway in treating SCI is apparent. The following summarizes recent therapeutic approaches targeting the Wnt signaling pathway in the context of SCI.

### 4.1 Glucocorticoids

Glucocorticoids currently stand as the sole pharmacological intervention directly employed in acute SCI. In rat SCI models, methylprednisolone activation of the Wnt/β-catenin signaling pathway has demonstrated effective neuronal protection (Lu et al., 2016). Notably, the impact treatments with methylprednisolone sodium succinate (MPSS) and methylprednisolone hemisuccinate (MF) in SCI induce downstream anti-inflammatory gene PPARγ expression within the Wnt/β-catenin pathway. This, in turn, inhibits pro-inflammatory factor levels, seemingly correlating with the extent of Wnt pathway activation (Libro et al., 2016). However, the side effects associated with glucocorticoid therapy should not be underestimated. Several studies have found that excessive glucocorticoids upregulate Wnt signaling pathway inhibitors, such as sFRP-1, DKK-1, and SOST, affecting skeletal structure and metabolism and potentially leading to osteoporosis (Mak et al., 2009; Nelson et al., 2019;). Additionally, excessive use of glucocorticoids in SCI has been shown to directly inhibit neuroregeneration in zebrafish (Nelson et al., 2019). Clinical debates persist regarding the therapeutic use of glucocorticoid drugs for SCI (Bowers et al., 2016; Evaniew et al., 2016). On one hand, glucocorticoids offer anti-inflammatory, antioxidant, edema-reducing, and membrane-stabilizing effects. On the other hand, their use may lead to a range of side effects, including increased risk of infection, gastrointestinal bleeding, and hyperglycemia (Pofi et al., 2023). Therefore, clinical application should be tailored to the patient’s specific conditions for a personalized treatment plan. In addition, the combined application of glucocorticoid drugs with other treatment strategies has demonstrated enhanced efficacy, including the use of nanomaterials (Chio et al., 2021; Lin et al., 2022). Given the substantial connection between glucocorticoid drugs and the treatment mechanisms of SCI, the exploration of combined interventions involving glucocorticoids and the Wnt signaling pathway remains limited. Nevertheless, such an approach holds promise in improving the therapeutic outcomes of SCI and mitigating complications.

### 4.2 Stem cell transplantation

Cell transplantation therapies hold potential for post-SCI repair and functional plasticity. Transplanted stem cells not only provide structural support and promote remyelination at the SCI site but also enhance the expression of neuroprotective factors, improve the spinal cord microenvironment, and facilitate the improvement of neural function (Zipser et al., 2022). Research indicates that the Wnt signaling pathway can enhance the proliferation and directional differentiation of neural stem cells (Wang Y. et al., 2019). In rat models of SCI, compared to the transplantation of solely neural stem cells, the overexpression of Wnt4 or Wnt5a in neural stem cells demonstrates a more efficient capability to promote neuronal differentiation, and it holds an advantage in motor function recovery following SCI (Li et al., 2015, 2020a,b; Chen J. et al., 2023).

Simultaneously, the Wnt signaling pathway significantly enhances the differentiation potential of stem cells from other sources, driving their differentiation into neurons and repairing the depleted neuronal population in the injury site. Transplantation of adipose-derived stem cells (ADSCs) has been proven as a safe and effective method for treating SCI (Duma et al., 2019; Tien et al., 2019). In *in vivo* studies, the involvement of Wnt3a and Wnt5a in regulating the neural differentiation of ADSCs has been identified, and activating the Wnt signaling pathway effectively enhances the restorative capacity of ADSCs in SCI (Yang et al., 2014). Furthermore, the chondroitin sulfate proteoglycan (CSPG) induced by SCI can impede the Wnt/β-catenin signaling pathway through the CSPG/LAR/PTPσ axis, diminishing the efficacy of neural stem cell transplantation (Hosseini et al., 2022). Therefore, achieving an appropriate level of activation in the Wnt signaling pathway is a crucial strategy to enhance the effectiveness of cell transplantation.

While stem cell transplantation therapy for spinal cord injuries holds immense clinical potential, it also faces numerous challenges (Chang et al., 2022). For instance, the survival rate of transplanted stem cells and their integration with host tissue pose significant hurdles (Liu et al., 2024). Transplanted stem cells need to survive within the injury’s microenvironment and effectively integrate into the host spinal cord neural network (Li G. et al., 2021). Stem cells possess a high proliferative capacity, which may entail the risk of tumor formation (Fujimori et al., 2012). Additionally, although animal models have shown promising results, the efficacy and long-term safety of stem cell therapy in human clinical trials require further validation.

### 4.3 Exosome therapy

Exosomes are small vesicles secreted by cells, with diameters ranging from 30 to 150 nanometers, playing a crucial role in intercellular communication. They regulate target cell functions by delivering various biomolecules, such as proteins, lipids, and RNA (including mRNA and non-coding RNA) (Feng et al., 2021). Exosomes are considered to have potential in promoting neural repair and regeneration. Firstly, they can modulate the immune response at the injury site by delivering immunoregulatory factors, reducing excessive inflammation, and thereby decreasing secondary damage to neural tissue (Zeng et al., 2023). Secondly, exosomes carry growth factors and neurotrophic factors such as brain-derived neurotrophic factor (BDNF) and neurotrophin-3 (NT-3), which can enhance neuron survival and regeneration, support axonal growth, and synaptic reconstruction (Yang et al., 2021; Chen Z. et al., 2023). Additionally, exosomes contain angiogenic factors like vascular endothelial growth factor (VEGF), which promote the formation of new blood vessels at the injury site, thereby improving local blood supply and nutritional support (Huang L. et al., 2022). Finally, exosomes can regulate the proliferation and differentiation of neural stem cells and progenitor cells, promoting their differentiation into functional cells such as neurons and oligodendrocytes, thereby facilitating remyelination after SCI (Li W. Y. et al., 2021).

SCI, exosomes from other stem cells (such as mesenchymal stem cells and neural stem cells) may promote neural repair and functional recovery by activating the Wnt/β-catenin signaling pathway (Liang et al., 2022). Research has found that exosomes derived from human umbilical cord mesenchymal stem cells (hUC-MSCs) activate the LRP-6/Wnt/β-catenin signaling pathway in neural cells after SCI, promoting the expression of c-myc and Cyclin D1 in spinal cord tissue, thereby exerting anti-apoptotic and anti-inflammatory effects to improve motor function (Kang and Guo, 2022). Additionally, exosomes from bone marrow mesenchymal stem cells (BMSCs-Exos) can activate the Wnt/β-catenin signaling pathway in neurons and inhibit neuronal apoptosis (Li et al., 2019). The extracellular vesicles originating from stem cells can significantly reduce the level of SCI damage by activating the Wnt/β-catenin pathway. Exosomes derived from M2 macrophages (M2-Exos) can activate the Wnt/β-catenin signaling in vascular endothelial cells at the mouse SCI site, positively regulating vascular regeneration and neural function repair (Luo et al., 2021).

Currently, researchers can extract exosomes from mesenchymal stem cells and neural stem cells, and modify exosomes through genetic engineering to increase the expression of Wnt proteins or regulatory factors, thereby enhancing their therapeutic efficacy. However, several challenges remain in their application. For instance, the production and purification processes of exosomes are complex and costly, and exosomes are prone to degradation during storage and transport, making large-scale production difficult. Another significant technical challenge is developing delivery systems that can ensure exosomes cross the blood–brain barrier, reach specific injury sites, and maintain sufficient concentrations locally. While exosomes hold great therapeutic potential for treating SCI, current research is still in the experimental stages using *in vitro* and *in vivo* models. Further efforts are needed to address these issues for successful clinical translation and application.

### 4.4 Biomaterials

Common biomaterials used for treating SCI include hydrogels, collagen, chitosan scaffolds, etc. These biomaterials not only provide a suitable environment for cellular and bioactive molecule interactions at the SCI site but also enhance therapeutic effects by binding with living cells, biomolecules, or other therapeutic agents (Han et al., 2022; Stropkovská et al., 2022; Ren et al., 2023; Sabourian et al., 2023).

Decellularized Extracellular Matrix Scaffolds (dECM) not only mimic the native three-dimensional structure of the spinal cord but also promote the release of neurotrophic factors and inhibit glial scar formation. Combined treatment with ADSCs, by upregulating the activity of the Wnt/β-catenin signaling pathway, demonstrates efficient neural repair potential (Su et al., 2023). Paclitaxel (PTX) induces neural stem cell differentiation into neurons by activating Wnt/β-catenin. A collagen microchannel scaffold can slow down PTX release, achieving sustained activation of the Wnt/β-catenin pathway in the SCI region for efficient repair (Chen et al., 2022). The fibrous hydrogel system, by controlling the sequential release of SDF1α and PTX, regulates the temporal window of action of the Wnt/β-catenin signaling pathway. This induces targeted migration of endogenous neural stem cells, enhances neuronal differentiation efficiency, and improves the therapeutic outcomes of SCI (Chen et al., 2022). Dental pulp stem cells/precursor cells (DPSCs), known for their high heterogeneity, have the potential to differentiate into neurons and glial cells (Young et al., 2016). When combined with chitosan scaffolds, DPSCs accumulate total β-catenin levels, inducing neuronal repair at the SCI site (Zhang et al., 2016).

In summary, the application of biomaterials not only provides a conducive environment for SCI repair but also controls the spatial and temporal effects of the Wnt signaling pathway. This helps reduce potential therapeutic risks and achieves high efficiency in SCI treatment.

### 4.5 Gene therapy

The Wnt protein family plays a critical role in regulating axonal preservation and regeneration following SCI. Utilizing gene therapy approaches, especially by modulating the Wnt signaling pathway, offers new prospects and strategies for treating SCI (Boato et al., 2023; Tan et al., 2024). By overexpressing and silencing target genes, it effectively suppresses damage such as inflammation, oxidative stress, and apoptosis, thus promoting neuronal regeneration. Thus far, *in vivo* gene therapy applications for SCI have primarily focused on enhancing the expression of regeneration factors, inhibiting the expression of harmful proteins, and introducing modifying enzymes to degrade inhibitory molecules (González et al., 2021; Ito et al., 2021; Voronova et al., 2022; Saijo et al., 2024).

Several studies have explored genetic engineering approaches to modulate the activity of the Wnt signaling pathway. For instance, increasing Wnt1 expression levels can improve motor function recovery in rats following spinal cord injury. However, Wnt1 protein itself cannot sustain high expression levels long-term in the injured spinal cord. Researchers employed lentiviral vectors to achieve sustained expression of the Wnt ligand, finding that prolonged overexpression of Wnt1 via lentivirus-mediated Fz1 ligand delivery enhances myelin preservation and neuronal survival, reduces early astroglial reactivity and accumulation of NG2+ cells, and improves motor function recovery in rats. This clearly supports inducing sustained high levels of Wnt1 expression during the progression of spinal cord injury as a therapeutic approach for treating motor dysfunction (González-Fernández et al., 2022). Another study found that the selective P2Y purinergic receptor agonist 2-MesADP can promote motor recovery following acute spinal cord injury. Researchers found that 2-MesADP exerts protective effects on local astrocytes at the injury site and induces the formation of reactive astrocytes. Additionally, it stimulates oligodendrocyte proliferation, providing a structural basis for neural signal transmission. Gene expression network analysis identified Nefh, NeuroD6, and Dcx as valuable potential targets in the 2-MesADP-treated group. It was confirmed that 2-MesADP regulates the expression of these genes through the Wnt signaling pathway, thereby inhibiting neuronal apoptosis, promoting myelin regeneration, and ultimately facilitating motor function recovery in mice with spinal cord injury (Zhao et al., 2020).

Gene therapy, as an emerging approach for treating spinal cord injury, holds significant potential but currently faces several major limitations and challenges. Firstly, most experiments are primarily conducted in animal models, and the critical question of how to effectively translate these findings into clinical practice needs careful consideration. Secondly, issues such as gene delivery, immune responses, safety, and efficacy must be further addressed to advance this field (Cunningham et al., 2023). Future studies should focus on optimizing gene delivery systems, enhancing the safety and effectiveness of treatments, and reinforcing clinical trials and regulatory frameworks to advance the application of gene therapy in SCI treatment.

### 4.6 Physical therapy

Physical therapy is an integral component of SCI rehabilitation, aiming to enhance traditional recovery treatments. Common physiotherapy modalities, such as electrical stimulation, magnetic stimulation, ultrasound stimulation, and light stimulation, have proven to be effective in functional recovery after SCI (Shao et al., 2021; Ghorbani et al., 2022; Neshasteh-Riz et al., 2022). These non-invasive treatments effectively increase neuronal excitability, improve blood circulation, and promote spinal cord functional recovery. Electrical stimulation, magnetic stimulation, and vibration stimulation can activate Wnt/β-catenin signaling in neural cells, enhancing the expression of brain-derived neurotrophic factor (BDNF), nerve growth factor (NGF), and vascular endothelial growth factor (VEGF), among other trophic factors. This promotes neuronal survival and functional recovery after SCI (Fang et al., 2021; González-Fernández et al., 2022; Huang J. H. et al., 2022). Thus, physical therapy can upregulate endogenous Wnt signaling expression, contributing to SCI repair.

While physical therapy is widely utilized in clinical treatment for SCI due to its non-invasive and simplicity approach, considering the complexity of SCI treatment, relying solely on physical therapy is insufficient. A comprehensive approach integrating pharmacotherapy, surgical interventions, stem cell therapy, gene therapy, and psychological support is necessary to provide patients with a comprehensive and systematic treatment plan, maximizing functional recovery and enhancing their quality of life. The combined application of these treatment modalities requires interdisciplinary collaboration, and personalized treatment plans tailored to each patient’s specific needs and circumstances, ensuring the scientific rigor and efficacy of the treatment regimen.

## 5 The Wnt signaling pathway in the context of other injuries

Wnts are critical regulatory factors involved in embryonic development and tissue differentiation, with almost negligible expression in adult tissues. However, they can be reactivated in the case of injury. The Wnt signaling pathway has been demonstrated to play a crucial role in the processes of injury and repair in the kidney, heart, liver, bone, and skin (Shen et al., 2021; Mensah et al., 2024; Oliva-Vilarnau et al., 2024; Wu et al., 2024). In adults, the Wnt pathway remains dormant and is reactivated upon injury (Aisagbonhi et al., 2011). In the initial minutes of acute injury, local vascular damage can induce platelet activation, participating in the primary hemostatic function. However, activation of the Wnt/β-catenin pathway in platelets can inhibit platelet activation and aggregation (Steele et al., 2009). The subsequent inflammatory response post-injury is not only beneficial for infection resistance and the clearance of dead cells but also induces mechanisms for repair. However, excessive inflammatory responses are detrimental to tissue repair (Ng, 2022). Generally, Wnt/β-catenin effectively inhibits inflammation, while the non-canonical Wnt signaling pathway may have pro-inflammatory effects. The balance between the two may modulate the levels of damage and repair (Zuriaga et al., 2017; Liu et al., 2023). In terms of tissue regeneration and repair, Wnt/β-catenin can induce mesenchymal transition, enhancing cell differentiation potential, thereby facilitating cell regeneration to repair the injury (Matsushita et al., 2020). Research also indicates that Wnt/β-catenin promotes hyaluronic acid formation, inducing fibrotic scar repair (Marinkovic et al., 2012). There is considerable controversy regarding the reparative role of Wnt signaling in tissue injury. Despite the activation of the Wnt signaling pathway in stem cells in response to tissue damage and its promotion of stem cell differentiation to replace damaged cells to some extent, there is evidence suggesting that some stem cells contribute to tissue repair by forming scar tissue, disrupting the original structure and function of the tissue (Liu et al., 2012, 2018; Wilson et al., 2020). In the repair of kidney injuries, the short-term and localized activation of the Wnt/β-catenin signaling pathway induces tissue regeneration and repair, while sustained activation leads to tissue fibrosis, chronic cell damage, and metabolic disorders (Pan et al., 2021; Guo et al., 2022).

In summary, the Wnt signaling pathway has demonstrated significant effects in the repair of various organs. Utilizing this pathway for treatment should involve comprehensive and precise approaches to develop more effective and safer therapeutic strategies for patients.

## 6 Conclusion

Currently, treating SCI remains a significant clinical challenge. As research advances, it has been found that the Wnt signaling pathway is involved in the proliferation and differentiation of neural stem cells, promotes axonal regeneration, modulates spinal inflammation, and inhibits neuronal apoptosis, bringing new hope for neural repair following SCI. However, there are several challenges in utilizing the Wnt signaling pathway for SCI treatment. Firstly, the Wnt pathway involves multiple signaling molecules and receptors, making it crucial to select appropriate binding sites to regulate the intensity and directionality of Wnt signaling. Secondly, despite the potential of the Wnt signaling pathway in promoting neural regeneration and repair, long-term therapeutic efficacy and safety still require further evaluation. Lastly, the translation from laboratory research to clinical application is a complex process that involves overcoming numerous technical, regulatory, and practical challenges. Ensuring the effectiveness and safety of treatment methods through validation in clinical trials is a critical step. Although the Wnt signaling pathway plays a crucial role in SCI repair, relying solely on a single intervention strategy may not be sufficient to effectively enhance recovery post-SCI. Future SCI treatments may require a combination of collaborative strategies, including the coordinated regulation of the Wnt signaling pathway with other signaling pathways, anti-inflammatory and immune modulation, stem cell therapy, tissue engineering and biomaterials, rehabilitation training and physical therapy, personalized and precision medicine, as well as interdisciplinary collaboration. By comprehensively applying these strategies, the efficacy of SCI treatment can be improved, promoting neural regeneration and functional recovery, thus enhancing patient prognosis and quality of life.

## Author contributions

KL: Conceptualization, Funding acquisition, Visualization, Writing – original draft, Writing – review & editing. ZC: Resources, Writing – original draft, Writing – review & editing, Methodology. XC: Visualization, Writing – original draft, Writing – review & editing, Resources. RX: Formal analysis, Visualization, Writing – original draft, Writing – review & editing. HW: Funding acquisition, Supervision, Writing – original draft, Writing – review & editing. WG: Funding acquisition, Supervision, Writing – original draft, Writing – review & editing.
